# Improving the identification of dementia with Lewy bodies in the context of an Alzheimer’s-type dementia

**DOI:** 10.1186/s13195-018-0356-0

**Published:** 2018-03-01

**Authors:** Alan J. Thomas, Fariba Mahin-Babaei, Mohammad Saidi, Debbie Lett, John Paul Taylor, Lauren Walker, Johannes Attems

**Affiliations:** Institute of Neuroscience, Newcastle University, Biomedical Research Building, Campus for Ageing and Vitality, Newcastle upon Tyne, NE4 5PL UK

**Keywords:** Dementia, DLB, AD, Neuropathology, Mixed pathology

## Abstract

**Background:**

Dementia due to Alzheimer’s disease (AD) and dementia with Lewy bodies (DLB) are the two most common neurodegenerative causes of dementia. They commonly occur together, especially in older people, but clinical identification of these diseases in dementia is difficult in such circumstances. We therefore conducted a study using cases with both comprehensive prospective clinical assessments and complete neuropathological examination to determine if it is possible to identify such mixed cases clinically and to determine features which may identify DLB in the presence of AD dementia.

**Methods:**

At Newcastle Brain Bank we identified subjects who had a clinical diagnosis of dementia and who also had autopsy diagnoses of pure AD, pure DLB, or mixed AD+DLB. All subjects had undergone prospective longitudinal clinical assessments. Mixed AD+DLB patients met neuropathological criteria for both DLB (limbic/neocortical Lewy body disease) and AD (Braak stage V/VI and CERAD B/C). The records of these subjects were carefully reviewed by two specialists in old-age psychiatry blind to autopsy findings to determine baseline and final clinical diagnoses based on these detailed records. The presence of characteristic Lewy body symptoms and other clinical information was also recorded.

**Results:**

Of 59 subjects included, 19 were AD, 18 DLB, and 22 mixed AD+DLB. At baseline no subjects were correctly identified as having mixed AD+DLB and by final diagnosis only 23% were identified. The only symptom which helped in identifying the presence of Lewy body disease in the context of a mixed AD+DLB dementia was complex visual hallucinations.

**Conclusions:**

Whilst the identification of DLB in the context of a dementia with an AD pattern is difficult, the emergence of complex visual hallucinations in the context of such a degenerative dementia suggests the presence of Lewy body disease and should encourage a careful assessment. Biomarkers appear likely to be necessary to help improve identification of different disease subtypes underlying dementia.

## Background

The presence of Lewy bodies (LBs) in the cortex of people with dementia was first reported by Okazaki et al. in two patients in 1961 [1] but it was the larger case series from Kosaka et al. about 20 years later [2] that established the link between dementia and cortical LB disease. Subsequent studies in the 1980s and 1990s led to the wider recognition of dementia with Lewy bodies (DLB) as a clinicopathological entity distinct from Alzheimer’s disease (AD) and further led to the formulation of the international consensus diagnostic criteria for DLB [3, 4]. These diagnostic criteria have a high specificity, but their sensitivity is considerably lower despite improvements in clinical diagnostics with the incorporation of REM sleep behaviour disorder (RBD) and FP-CIT imaging [5, 6]. Hence, many DLB patients are clinically misdiagnosed as AD and therefore not provided with appropriate treatment.

A few previous clinicopathological studies have endeavoured to determine a clinical correlate that that will help identify the clinical manifestation of DLB in the presence of co-morbid AD [7–10]. Whilst suggesting that visuospatial dysfunction or visual hallucinations may help identify DLB in the context of an AD-type dementia, these studies have had low numbers (e.g. [8]), limited clinical assessments, and did not fulfil the full complement of consensus criteria required for definite AD and DLB [4, 11–14].

It therefore remains unclear whether it is possible for clinicians to detect the presence of DLB by clinical assessment in the context of a dementia that is also due to AD, and if so what features might enable such identification. The clinical importance of identifying DLB to avoid inappropriate and dangerous use of antipsychotics is now well recognised [15], but missing people who develop DLB in the context of AD dementia puts people at risk of such adverse reactions. Furthermore, the prospect of disease-modifying drug therapies has encouraged the move towards identification of specific diseases and stratification of dementia subgroups by aetiology. The development of any successful disease-modifying treatments for amyloid, tau, or synuclein pathology would make the identification of co-morbidity highly important to facilitate stratification for treatment optimisation, especially as it has been demonstrated that co-morbid tau pathology confers a worse prognosis in patients with DLB [16]. We therefore investigated firstly whether specialist old-age psychiatrists could distinguish autopsy-confirmed mixed AD+DLB cases from those clinically and neuropathologically diagnosed as pure AD or pure DLB, and secondly what clinical features might enable such a distinction to be made.

## Methods

### Study cohort

We searched the Newcastle Brain Tissue Resource (NBTR) and identified 22 cases that fulfilled neuropathological criteria for both full-blown AD and DLB, i.e. mixed AD+DLB. In addition, 19 cases were included that fulfilled neuropathological criteria for pure AD and 18 for pure DLB. Demographic data of our study cohort are displayed in Table 1. Cases came to autopsy between 2005 and 2015 and the AD subjects made up 20% of those in our brain bank; the DLB subjects made up 60%. All cases had longitudinal clinical data available that were retrieved from relevant research records and all secondary care health service records, detailing assessments, investigations, and management in memory/dementia services and all other health services (see below). All these clinical records were assessed by two specialists in old-age psychiatry (FMB and MS) who were blind to the autopsy findings.

**Table 1:** Characteristics of subjects by final neuropathological diagnosis

	AD	DLB	Mixed AD+DLB	
	*N* = 19	*N* = 18	*N* = 22	
Age at baseline (years)	76.8 (9.3)	71.0 (6.1)	70.5 (9.0)	F = (2,56) 3.4, *P* = 0.04
				DLB vs AD, *P* = 0.03
				DLB vs mixed, *P* = 0.87
				AD vs mixed, *P* = 0.04
Age at death (years)	82.7 (9.4)	76.9 (5.3)	78.2 (8.8)	F = (2,56) 2.6, *P* = 0.08
				DLB vs AD, *P* = 0.03
				DLB vs mixed, *P* = 0.58
				AD vs mixed, *P* = 0.16
Gender (male:female)	13:6	14:4	16:6	X = 0.41, df = 2, *P* = 0.82
Interval between baseline and death (years)	5.8 (2.5)	5.9 (1.9)	7.8 (2.6)	F = (2,56) 4.5, *P* = 0.015
				DLB vs AD, *P* = 0.83
				DLB vs mixed, *P* = 0.02
				AD vs mixed, *P* = 0.02
Baseline MMSE	18.0 (7.5)	25.3 (2.7)	22.0 (3.9)	F = (2,52) 8.5, *P* = 0.001
				DLB vs AD, *P* = 0.001
				DLB vs mixed, *P* = 0.01
				AD vs mixed, *P* = 0.04
Final MMSE	5.9 (7.8)	12.8 (6.9)	6.8 (9.4)	F = (2,53) 3.6, *P* = 0.04
				DLB vs AD, *P* = 0.01
				DLB vs mixed, *P* = 0.04
				AD vs mixed, *P* = 0.75
Interval between last assessment and death (months)	20.9 (13.8)	25.4 (17.1)	35.9 (28.7)	F = (2,51) 2.5, *P* = 0.09
				DLB vs AD, *P* = 0.40
				DLB vs mixed, *P* = 0.19
				AD vs mixed, *P* = 0.05

*AD* Alzheimer’s disease, *DLB* dementia with Lewy bodies, *MMSE* Mini-Mental State Examination

### Clinical assignment of diagnoses

Assessment involved the specialists carefully reading all health service and research records, noting key symptoms, and summarising the data. Following this, each specialist then assigned baseline and final clinical diagnoses to all patients whilst blind to autopsy findings based on this information. Baseline diagnosis was that at the first recorded clinical assessment and final diagnosis was that made at the last time before death when all relevant clinical information was available (service records on terminal illness did not usually provide information relevant to making or altering a dementia subtype diagnosis). Where there was disagreement about the diagnosis (which occurred in 11 cases at baseline and 14 at final diagnosis, overall 21% of cases) a third independent expert in dementia diagnosis (AT) reviewed the records to assign a consensus final clinical diagnosis. Pure (p)AD and pDLB cases were diagnosed by applying standard diagnostic criteria (NIA-AA (2011) [17] and Consensus DLB Third report (2005) [3]). At baseline, the presence of any supportive LB features, e.g. delusions or depression, in a case (without core LB symptoms) raised the possibility of it being mixed. After baseline, the emergence of core LB symptoms or supportive symptoms in someone with gradual cognitive decline and worsening functional impairment (i.e. AD-like decline) raised the possibility of this case being mixed.

To ensure there was consistency in the identification of relevant clinical data, each specialist independently reviewed records on six patients who had been reviewed by the other specialist. Their records were compared and there was good agreement on the data obtained.

### Neuropathological diagnosis

At autopsy the right hemisphere, brainstem, and cerebellum were immersed and fixed in 4% buffered aqueous formalin for 4–6 weeks. Brain regions including frontal, temporal, parietal and occipital cortices, cingulate and hippocampus, striatum (including both caudate nucleus and putamen), amygdala, midbrain, and locus coeruleus were embedded into paraffin wax, sectioned at 6 μm and mounted on 4% 3-aminopropyltriethoxysilane slides.

Immunostaining with monoclonal antibodies against hyper-phosphorylated tau (HP-τ; AT8, dilution 1:4000, Innogenetics, Ghent, Belgium), Aβ (4G8, dilution 1:15,000, 4G8, Signet Labs, Dedham, MA, USA), and α-synuclein (α-syn, dilution 1:200, Chemicon, Hofheim, Germany) was carried out. Prior to this, slides were microwaved for antigen retrieval in 0.01Mol L-1 citrate buffer for 10 min (AT8), pressure cooked in 0.01Mol L-1 EDTA for 90 s (α-syn), or immersed for 1 h in Formic acid (4G8). Immunopositivity was assessed using a MENAPATH HRP polymer detection kit (Menarini diagnostics, Berkshire, UK) with chromagen 3,3 diaminobezidine (DAB) and counterstained with haematoxylin. Tissue was finally dehydrated through an alcohol series, cleared, and mounted using DPX (CellPath, Powys, UK).

Pathological diagnoses were assigned using internationally accepted criteria by a senior neuropathologist (JA). This included neurofibrillary tangle (NFT) Braak stages [11, 18], CERAD scores [13], NIA-RI criteria, where Thal amyloid β phases were available NIA-AA criteria [12, 14, 19], and the Newcastle McKeith criteria for DLB [3, 4]. Subjects were divided into three groups, pure AD (pAD), pure DLB (pDLB), and mixed AD+DLB, the latter being assigned when the neuropathological criteria was fulfilled for both AD and DLB [20]. Subjects assigned to the pAD and pDLB groups displayed only minimal concomitant pathology associated with other neurodegenerative diseases or cerebrovascular disease.

### Statistical analysis

The Statistical Package for Social Sciences software (SPSS version 23) was used for statistical evaluation. For group comparisons, chi-squared tests were used for categorical variables and for continuous variables we tested for normality of distribution and analysis of variance (ANOVA) and *t* tests or Mann Whitney tests were used. Clinician diagnostic accuracy (sensitivity, specificity, and overall accuracy) was calculated from standard 2 × 2 frequency tables and 95% confidence intervals for these calculated using Minitab (version 16.1). Inter-rater reliability was assessed using the kappa coefficient.

The tissue and associated data used in this study were from the NBTR and used in accordance with ethical approvals for NBTR.

## Results

### General observations

Study demographics are displayed in Table 1. Patients with a neuropathological diagnosis of mixed AD+DLB and those diagnosed as pDLB were significantly younger than pAD patients at baseline clinical assessment (*P* < 0.05). Patients with a neuropathological diagnosis of mixed AD+DLB had a significantly longer time interval between baseline clinical assessment and death compared to both pAD and pDLB (*P* < 0.05). At baseline, the Mini-Mental State Examination (MMSE) score in pAD cases was significantly lower than in the mixed AD+DLB group (*P* < 0.05), while such a difference was not observed at final assessment (*P* = 0.75). Although not statistically significant, the interval between last clinical assessment and death was longer in the mixed AD+DLB group compared to the pAD (*P* = 0.05) and pDLB (*P* = 0.19) groups. Controlling for such differences did not affect any of the findings below.

### Baseline clinical diagnoses

At baseline none of the subjects who had mixed AD+DLB pathology were diagnosed as such. Each had been given a specific subtype diagnosis. Thus, sensitivity of clinical diagnosis for neuropathologically mixed AD+DLB at baseline was 0% and specificity was 100%, giving an overall diagnostic accuracy of 62.7%. For pAD the sensitivity was 74% (95% confidence interval, 49 to 91), the specificity was 63% (95% confidence interval, 46 to 77), and the overall diagnostic accuracy was 66% (95% confidence interval 53 to 78), and for pDLB the sensitivity was 89% (95% confidence interval 65 to 99), the specificity was 78% (95% confidence interval 62 to 89), and the overall diagnostic accuracy was 81% (95% confidence interval 69 to 90). The kappa coefficient for inter-rater reliability at baseline assessments was 0.56.

### Final clinical diagnosis

At final clinical assessment ten individuals were assigned a clinical diagnosis of mixed AD+DLB, however only five of these of these fulfilled neuropathological criteria for mixed AD and DLB at *post-mortem* examination. Of the 22 neuropathologically diagnosed mixed AD+DLB cases, the remaining seventeen were either given a clinical diagnosis of AD (*n* = 7) or DLB (*n* = 10). Thus, the sensitivity for mixed AD+DLB was only 23% (95% confidence interval, 8 to 45) although the final specificity was 87% (95% confidence interval, 71 to 96), giving an overall diagnostic accuracy of 63% (95% confidence interval, 49 to 75). For the pAD cases final sensitivity was 68% (95% confidence interval, 43 to 87) and the specificity was 80% (95% confidence interval, 64 to 91) (overall accuracy 76% (95% confidence interval, 63 to 86)) and for pDLB it was 89% (95% confidence interval, 65 to 99) and 71% (95% confidence interval, 45 to 89), respectively (overall accuracy 76% (95% confidence interval, 63 to 86)). The kappa coefficient for inter-rater reliability for final assessments was 0.59.

### Individual symptoms

We also explored whether core, suggestive, and supportive symptoms of DLB might individually be useful to clinically identify neuropathologically mixed AD+DLB cases (see Table 2). As expected, three key LB symptoms, complex visual hallucinations (VH), parkinsonism, and RBD, had different frequencies between the groups. At baseline, VH were less frequent in the mixed AD+DLB cases compared to pDLB (*P* < 0.01), with no difference between mixed AD+DLB and pAD. However, 50% of mixed AD+DLB cases went on to develop VH during the time course of the disease, which was significantly higher than the pAD group (*P* < 0.05), although still lower than the pDLB group (*P* < 0.01). At baseline, the prevalence of RBD in neuropathologically mixed AD+DLB cases was significantly lower than in pDLB cases (*P* < 0.01) and was not different from the pAD group (*P* = 0.46), which was similar when assessing the groups for the emergence of RBD at any point for the disease duration. Whilst parkinsonism occurred in more people with mixed AD+DLB than in those with pAD this was not statistically different. Correcting for differences in age and dementia severity (MMSE score) did not affect this finding. Similarly, cognitive fluctuations and neuroleptic sensitivity were identified in similar proportions in mixed AD+DLB cases and pAD cases, rendering these features not useful for identifying mixed AD+DLB cases. None of the other clinical features examined approached significant differences (see Table 2).

**Table 2:** Lewy body symptoms by final neuropathological diagnosis

	AD	DLB	Mixed AD+DLB	
	*N* = 19	*N* = 18	*N* = 22	
Core and suggestive symptoms				
Cognitive fluctuations ever	5 (26%)	9 (50%)	8 (36%)	X = 2.23, df = 2, *P* = 0.33
Complex visual hallucinations at any time	3 (16%)	17 (94%)	11 (50%)	X = 23.0, df = 2, *P* < 0.001
				AD vs mixed, X = 5.31, df = 1, *P* = 0.02
				DLB vs mixed, X = 9.30, df = 1, *P* < 0.01
Complex visual hallucinations at baseline	1 (5%)	12 (67%)	3 (14%)	X = 20.86, df = 2, *P* < 0.01
				AD vs mixed, X = 0.81, df = 1, *P* = 0.36
				DLB vs mixed, X = 11.88, df = 1, *P* < 0.01
Complex visual hallucinations emerging later	2 (11%)	5 (28%)	8 (36%)	X = 3.67, df = 2, *P* = 0.16
				AD vs mixed, X = 3.69, df = 1, *P* = 0.055
				DLB vs mixed, X = 0.33, df = 1, *P* = 0.564
Parkinsonism at any time	4 (21%)	12 (67%)	10 (45%)	X = 7.83, df = 2, *P* = 0.02
				AD vs mixed, X = 2.7, df = 1, *P* = 0.1
				DLB vs mixed, X = 1.80, df = 1, *P* = 0.15
Parkinsonism at baseline	1 (5%)	8 (44%)	5 (23%)	X = 7.86, df = 2, *P* = 0.02
				AD vs mixed, X = 2.49, df = 1, *P* = 0.13
				DLB vs mixed, X = 1.36, df = 1, *P* = 0.2
Parkinsonism emerging later	3 (16%)	4 (22%)	5 (23%)	X = 0.36, df = 2, *P* = 0.84
RBD at any time	4 (21%)	12 (67%)	7 (32%)	X = 7.83, df = 2, *P* = 0.02
				AD vs mixed, X = 0.60, df = 1, *P* = 0.34
				DLB vs mixed, X = 4.82, df = 1, *P* = 0.03
RBD at baseline	1 (5%)	6 (33%)	0 (0%)	X = 11.69, df = 2, *P* < 0.01
				AD vs mixed, X = 1.19, df = 1, *P* = 0.46
				DLB vs mixed, X = 8.63, df = 1, *P* < 0.01
RBD emerging later	3 (16%)	6 (33%)	7 (32%)	X = 1.83, df = 2, *P* = 0.4
Neuroleptic sensitivity at any time	3 (16%)	2 (11%)	3 (14%)	X = 0.17, df = 2, *P* = 0.92
Exposed to use of neuroleptic	7 (37%)	9 (50%)	12 (55%)	X = 1.35, df = 2, *P* = 0.51
Supportive symptoms				
Falls at any time	7 (37%)	9 (50%)	11 (50%)	X = 0.90, df = 2, *P* = 0.64
Other hallucinations at any time	1 (5%)	2 (11%)	5 (23%)	X = 2.79, df = 2, *P* = 0.25
Delusions at any time	3 (16%)	7 (39%)	6 (27%)	X = 2.79, df = 2, *P* = 0.25
Depression at any time	10 (53%)	9 (50%)	6 (27%)	X = 3.30, df = 2, *P* = 0.19
Anxiety at any time	11 (58%)	9 (50%)	11 (50%)	X = 0.32, df = 2, *P* = 0.85
Behavioural disturbance at any time	11 (58%)	8 (44%)	12 (55%)	X = 0.73, df = 2, *P* = 0.70

*AD* Alzheimer’s disease, *DLB* dementia with Lewy bodies, *RBD* REM sleep behaviour disorder

### Mixed cases

Of the 22 neuropathologically mixed AD+DLB cases, eight had a baseline diagnosis of DLB and all of these were regarded as having clinical DLB as their final diagnosis. Of the 14 who had AD as the baseline diagnosis, seven subsequently had AD as their final diagnosis and none of these had any identifiable LB symptoms at any stage. The other seven did develop LB symptoms and five of these were diagnosed as mixed AD+DLB while the other two had a final diagnosis of DLB. In these latter two cases the gradual onset of degenerative dementia was regarded as being due to DLB with LB symptoms emerging later which enabled differentiation from AD.

Since these findings in the mixed group suggest that the emergence of LB symptoms in the context of an AD presentation should raise concerns about the presence of LB disease, we compared these with the 19 pure AD cases. Two of these 19 cases appeared also to later develop VH. One had VH but importantly these were not the complex VH in the DLB diagnostic criteria, but simple fleeting hallucinations, which often are associated with poor eyesight. In the other case the patient had an initial paranoid psychosis and developed parkinsonism on antipsychotics as well as a dementia. He also had an abnormal ^123^I-FP-CIT SPECT dopaminergic imaging scan which was thought clinically and at autopsy to be due to brainstem cerebrovascular disease.

## Discussion

We found that identification of DLB in the context of an AD pattern of dementia is difficult but that complex visual hallucinations emerging later in the course of an AD-type dementia suggest the presence of additional LB disease pathology and, thus, mixed AD+DLB. This finding is consistent with other studies, though importantly these did not assess mixed cases [7–10]. In such circumstances a careful review of the diagnosis is merited, including scrutiny for other symptoms of LB disease, and antipsychotic medication should be avoided.

At baseline none of the mixed AD+DLB cases was identified and even at final assessment, with full information on the evolution of the dementia, sensitivity for detecting mixed AD+DLB was only 23%. Whilst we found that the emergence of complex VH suggests the presence of additional LB disease pathology and thus mixed AD+DLB, no other DLB features proved useful for identifying the presence of LB disease in the mixed AD+DLB group in our study. Although parkinsonism occurred more often in people who had mixed AD+DLB this was not significantly greater than in AD, especially later on in the illness where involvement of the substantia nigra by tau pathology can cause parkinsonism [18]. However, the likelihood that parkinsonism in the context of AD may be due to vascular causes at baseline (see the end of the Results section) suggests that the presence of parkinsonism due to Parkinson’s disease may be a useful feature in identifying mixed AD+DLB if this can be reliably identified. Similarly, RBD occurred more frequently in mixed AD+DLB but not significantly more often than in AD, especially later. It is surprising that several pAD cases were identified as having RBD since this feature is highly specific for LB disease and these are likely to have been false positives, a finding that is probably related to the difficulty in accurately identifying RBD in people with more severe dementia where reliable clinical information can be difficult to obtain. Overall, although RBD and parkinsonism appeared to emerge in a few pAD subjects, we are uncertain about the validity of these symptoms and do not regard these in the context of more advanced dementia as symptoms which would be useful in supporting a DLB diagnosis. Although RBD is highly specific to synucleinopathies it is difficult to reliably identify it clinically, and parkinsonism is both difficult to identify clinically and may be due to other pathologies, especially in more severe dementia.

One reason for the ‘failure’ to identify symptoms is that the secondary pathology may not be present or advanced enough to be detected clinically at the early disease stage, since study subjects were only at the mild dementia stage at baseline assessment. Alternatively, the secondary pathology may be present but clinical assessment alone is not sensitive enough to detect this and biomarkers are necessary to do so. The absence of such biomarker data is a limitation of our study, though one which reflects the period in which cases were assessed. There are two biomarkers validated against autopsy and of proven diagnostic value in distinguishing DLB from AD, dopaminergic imaging (specifically, FP-CIT SPECT imaging [21]) and cardiac MIBG imaging [22]. None of our subjects had MIBG since this is rarely used in the United Kingdom, and only 16 had FP-CIT dopaminergic scans. These cases were spread across the groups so that the numbers were too low for meaningful comparison. None of our patients had *in vivo* amyloid or tau imaging and such assessments may make it possible in future to identify AD in such mixed cases. Preliminary studies investigating the tau ligand ^18^F-AV-1451 have indicated increased binding of ^18^F-AV-1451 in the medial temporal lobe can distinguish AD from probable DLB patients [23], and elevated cortical binding of ^18^F-AV-1451 to tau in DLB patients is associated with a decline in cognition [24]. Furthermore, a quantitative *post-mortem* pathological study has demonstrated a higher hyper-phosphorylated tau burden in neuropathologically mixed AD+DLB cases that had a clinical presentation of AD compared with those with a DLB phenotype [20]. This may indicate that these cases were originally following the typical course of AD and developed LB pathology later. Currently there are no synuclein-specific ligands for in vivo imaging to identify LB disease. In the future the use of such disease-specific imaging is likely to aid differential diagnosis and disease stratification for clinical trials, though currently the cost of such scans prohibits widespread use and it is likely that criteria will be needed to target such scans that will include identifying clinical features such as complex visual hallucinations that support the presence of LB disease.

When we focussed our analysis on the neuropathologically mixed AD+DLB cases this helped to further elucidate the difficulty in identification of LB disease in such patients. About one-third (8 of 22) of the patients appeared to begin their illness with clinical features consistent with DLB. Such cases may have had co-AD pathology at this time or may subsequently have developed AD pathology, but since no specific AD symptoms allow its identification and no amyloid or tau biomarkers were used in this study then such AD could not be identified clinically. Another third (7 of 22) of the neuropathologically mixed AD+DLB cases had a typical AD presentation with no features suggestive of LB disease at all. The final third of neuropathologically mixed AD+DLB cases did have clear core and/or suggestive features of DLB, but in these cases DLB-associated symptoms emerged later. These findings are consistent with previous reports that high-level Alzheimer’s pathology masks the additional presence of characteristic LB symptoms and thus the manifestation of LB disease [25]. Specifically, it is not amyloid burden [26] but tau pathology burden that obscures the presence of additional LB disease [27].

Clinico-pathological studies have endeavoured to determine a clinical correlate that that will best predict the clinical manifestation of DLB in the presence of co-morbid AD. Comparison with these earlier studies is limited by the different criteria being used such that, for example, Lewy Body Variant (LBV) of AD was a broader category including people with lower Braak stages than in our study. Tiraboschi and colleagues found that visual hallucinations were a highly specific feature for differentiating DLB and AD in a neuropathologically validated cohort of AD and DLB [7], and we have now shown that the development of such hallucinations in people with an AD pattern of dementia indicates the presence of DLB co-pathology, that is a mixed AD+DLB dementia. An early study by Hansen et al. found visuospatial dysfunction, deficits in verbal fluency and attention, and mild parkinsonian features to be more prominent in patients with mixed AD+DLB pathology (termed by the authors as Lewy body variant of AD) than in patients showing AD pathology only [8]. However, this study was limited by a relatively low sample size (nine mixed AD+DLB), the use of ubiquitin immunolabelling rather than α-synuclein for the detection of LB, and the absence of a pure DLB group for comparison. Another study found a higher frequency of visual hallucinations in mixed AD+DLB cases compared to pure AD cases, but failed to detect clinical differences between mixed AD+DLB and pure DLB [9]. This study used α-synuclein immunohistochemistry for some subjects but had limited ascertainment of important clinical symptoms (e.g. visual hallucinations or cognitive fluctuations) as these were not specifically clinically assessed. They did find, however, that extrapyramidal symptoms were more frequent in the mixed AD+DLB group but this was confounded by the large proportion of patients (about two-thirds) on antipsychotic drugs. A more recent study using α-synuclein labelling in all cases compared autopsy-confirmed mixed AD+DLB to classical AD and DLB cases in patients with mild dementia at their first clinical assessment [10]. This study included detailed neuropsychological assessment of patients but less thorough clinical evaluation of characteristic DLB symptoms. It found expected differences between classical AD and classical DLB groups (worse visuospatial function and better memory, as well as more extrapyramidal symptoms in DLB), but no differences were identified between the mixed AD+DLB patient group and the AD group. In addition, it is important to note that neuropathological assessment in the aforementioned studies did not fulfil the full complement of consensus criteria required for definite AD and DLB [4, 11–13, 19]. For example, the study by Yoshizawa and colleagues [10] contained cases with neurofibrillary tangle (NFT) Braak stage IV in their AD and mixed AD+DLB groups whilst their DLB group contained cases with NFT Braak stage III, both of which would be categorised as having intermediate AD neuropathologic change according to the National Institute on Ageing—Alzheimer’s Association criteria [19].

Another point to consider is the putative interactions between multiple pathologies and the cumulative effect this exerts on clinical dementia. Studies have demonstrated that co-morbid pathologies are common in DLB [28–31]; in particular, AD neuropathology has been shown to associate with the timing and onset of dementia in Lewy body diseases [16, 32]. Furthermore α-synuclein has been shown to promote the aggregation of both hyper-phosphorylated tau and β amyloid [33, 34], which in the context of LB disease may enhance clinical features associated with AD and mask the DLB phenotype.

The prevalence of DLB varies depending on the type of study and provenance of the patients, with brain bank studies reporting DLB as constituting 12.5% of dementia, hospital clinical studies indicating 7.2%, and community studies reporting only 4.2% [35]. Whilst some of the differences in these figures may result from selection bias, other factors are also important. Some of these ‘missing’ DLB cases seem to reflect clinical experience and thus expertise in diagnosis, with one study reporting variation amongst secondary care settings in England ranging from DLB constituting 2% of diagnosed dementia cases to DLB making up 8% of dementia, a finding not explainable by regional variation or service type [36]. However, in ADNI, subjects were thoroughly assessed and diagnosed with either clinical dementia due to AD or mild cognitive impairment (MCI) due to AD, and yet at autopsy DLB was found in 45.5% of cases [37]. This finding again demonstrates the high prevalence of unidentified LB disease even in a thoroughly assessed AD cohort. Others have reported that about 50% of AD cases have significant LB pathology [38]. Approaching this from the DLB diagnosis end, then perhaps 80% of such cases have some AD co-pathology [3]. It is important to distinguish between mixed pathology [31], which includes all different degrees of severity, and mixed dementia, which only refers to cases which show the full-blown pathology of two (or more) diseases such as mixed AD+DLB [20, 39]. The number of ‘missed’ LB cases depends on such definitions. However, our study suggests that whilst many of these ‘missing LB cases’ cannot be identified, at least without specific biomarkers, careful assessment for the presence of complex visual hallucinations would help in identifying many such cases.

This study benefited from having a thorough diagnostic review by specialist doctors using all available clinical and research records, and by the comprehensive neuropathological assessments of all cases. Although our subjects had detailed clinical and research records available, recording the prospective development of symptoms in their dementia, they were not all specifically prospectively reviewed in a research study designed to identify LB symptoms and so we cannot be sure that all relevant symptoms were assessed at reviews. It should also be noted that the mean time interval between final clinical assessment and death in the neuropathologically mixed AD+DLB group was just over 3 years, therefore giving time for progression of LB pathology and clinical expression of associated symptoms prior to neuropathological assessment. Furthermore, we have discussed the absence of biomarkers in this study above, reflecting the era in which our brain bank subjects were assessed.

## Conclusions

Careful evaluation for complex visual hallucinations can help identify the presence of Lewy body disease in the context of a dementia with a gradual decline typical of AD. Such recognition of mixed AD+DLB may improve clinical care by optimising DLB treatment in this context. The recognition of multiple pathologies underlying a dementia will become even more important when disease-modifying treatments become available which target specific pathologies, e.g. amyloid deposition. The use of biomarkers appears likely to be necessary for such disease identification.

## Abbreviations

AD: Alzheimer’s disease
DLB: Dementia with Lewy bodies
LB: Lewy body
MMSE: Mini-Mental State Examination
NBTR: Newcastle Brain Tissue Resource
NFT: Neurofibrillary tangle
p: Pure
RBD: REM sleep behavior disorder
VH: Visual hallucinations
